# Electrical Interference Simulation and Prediction Model for Acoustoelectric Logging Detector

**DOI:** 10.3390/s23083928

**Published:** 2023-04-12

**Authors:** Hongzhi Chen, Junqiang Lu, Xiaodong Ju, Baiyong Men, Wenxiao Qiao

**Affiliations:** 1 State Key Laboratory of Petroleum Resources and Prospecting, China University of Petroleum (Beijing), Beijing 102249, China; 17801232800@163.com (H.C.); juxdong@cup.edu.cn (X.J.); mby08@126.com (B.M.); qiaowx@cup.edu.cn (W.Q.); 2 Key Laboratory of Earth Prospecting and Information Technology, Beijing 102249, China

**Keywords:** acoustoelectric logging, acoustoelectric logging detector, high-voltage pulse, capacitive coupling, electrode measurement loop

## Abstract

Acoustic logging instruments generate high voltages in the order of thousands of volts. Electrical interferences are thus induced by high-voltage pulses that affect the logging tool and make it inoperable owing to damaged components in severe cases. High-voltage pulses from the acoustoelectric logging detector interfere with the electrode measurement loop through capacitive coupling, which has seriously affected the acoustoelectric signal measurements. In this paper, we simulate high voltage pulses, capacitive coupling and electrode measurement loops based on qualitative analysis of the causes of electrical interference. Based on the structure of the acoustoelectric logging detector and the logging environment, an electrical interference simulation and prediction model was developed to quantify the characteristics of the electrical interference signal.

## 1. Introduction

In the field of oil and gas exploration, the electromagnetic waves generated by the excitation of acoustic waves and the effect of coupling and conversion between the wave fields are referred to as the Acoustoelectric Effects [1,2]. These theoretical and experimental studies [3,4,5,6,7] have demonstrated that acoustoelectric logging can be directly applied to detecting formation properties related to pore fluid, such as conductivity, porosity, viscosity, ion concentration, and permeability. In particular, the formation permeability can be detected directly by acoustoelectric logging, and most logging methods cannot achieve that function. AcoustoElectric Logging Tool 2.0 (AELT 2.0) is a second-generation acoustoelectric logging detector [8,9,10] developed by the Acoustic Logging Laboratory of China University of Petroleum (Beijing).

Electrodes received a large interference signal at the zero moment, which was incidental to the work of the transmitting acoustic system. When logging, the interface acoustoelectric conversion wave arrives early, about 10 µs [11]. At this time, the amplitude of the interference is considerably larger than the interface acoustoelectric conversion wave, which causes it to be drowned. If the interference lasts long, it will mask the later accompanying acoustoelectric conversion wave signals, distorting the data. It can be seen that the interference has seriously jeopardized the acoustoelectric measurements.

At present, the progress of research on electrical interference of acoustoelectric logging detectors is mainly a summary of experiments. According to Sun, electrical interference is generated by the radiation of high-voltage pulse source [12]. According to Yin, electrical isolation of the transmitting and receiving circuits is not effective against electrical interference [13]. In the experiments of Zheng, the longer the trailing of the high voltage pulse, the longer the electrical interference. By adding impedance matching circuit to the transducer, the electrical interference is significantly shorter [14]. According to Fu and Wang, the electrical interference is related to the source excitation parameters, such as excitation pulse width, excitation power and source location [15,16]. According to Sheng, electrical interference arises from sudden voltage changes on the surface of the transmitting transducer [17].

By qualitatively analyzing the main interference causes of the acoustoelectric logging detector, an electrical interference simulation and prediction model based on AELT 2.0 and the logging environment was developed to quantitatively simulate the electrical interference caused by high voltage pulses to the electrode measurement loop through capacitive coupling. The analysis of the coupling path of the detector is more complex, which is related to the system structure, circuit layout and wiring. Changes in the resistivity of the drilling fluid and the formation can also have an effect on the electrode measurements. Thus, the model is able to describe both the electrical interference characteristics of AELT 2.0 and the response of electrical interference in the logging environment.

The interference signal not only affects the observation of the acoustoelectric signal, but it also leads to the inability to obtain clean results by data processing. The electrical interference simulation and prediction models not only assesses the degree of influence of interference signals on acoustoelectric signals and judges the effectiveness of logging data, but also predicts the effect of EMC rectification.

## 2. Analysis of the Causes of Electrical Interference

### 2.1. Structure of the Detector

**Figure 1:**
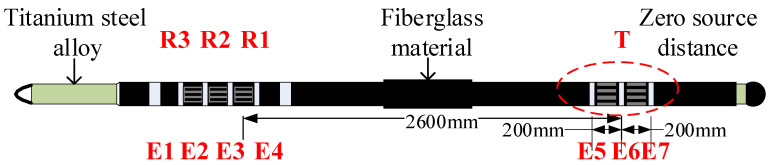
AELT 2.0.

The structure of the AELT 2.0 [18] is shown in Figure 1. The transmitting transducer T is a binary linear phased array. Each array element is composed of three high-power monopole transmitting transducers connected in parallel, generating 3800 V in a certain delay sequence. The high-voltage pulse excites the transmitting transducer. Four measuring electrodes, E1–E4, are added around the receiver transducer R1, R2, and R3. The instrument adds three measuring electrodes, E5, E6 and E7, around the transmitting transducer T. The distance between the electrodes is 200 mm. E6 is received in the form of potential, and the combination of E5 and E7 is a differential mode receiver that realizes the measurement of the interface acoustoelectric conversion wave signal at the zero-source distance. For the insulation between the electrodes, fibreglass material is used for the casing of the short section of the composite detector, while titanium steel alloy is used for the casing of the main control circuit and reference electrode.

The auxiliary measurement electrodes, E5, E6 and E7, acquire the interface acoustoelectric conversion wave near the sound source, whose energy is highly correlated with the acoustoelectric coupling coefficient and even the formation permeability, which is one of the important features added to AELT 2.0 compared to AELT 1.0.

### 2.2. Interference Source

**Figure 2:**
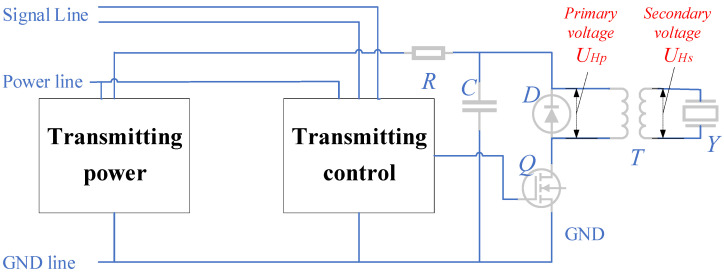
Working principle of the transmitting acoustic system.

The high-voltage pulses generated by the transmitting circuit are the main source of interference in the system. The operating principle of the transmitting acoustic system [19] is shown in Figure 2. The transmitting power charges the high-voltage capacitor C. When the system receives a discharge command, the transmitting circuit provides a trigger pulse to transistor Q to turn it on. Thus, C discharges through the discharge circuit composed of the primary coil T and the ground line. Finally, a secondary voltage $U_{Hs}$ is obtained on the secondary coil to excite the transducer Y.

### 2.3. Sensitive Source

**Figure 3:**
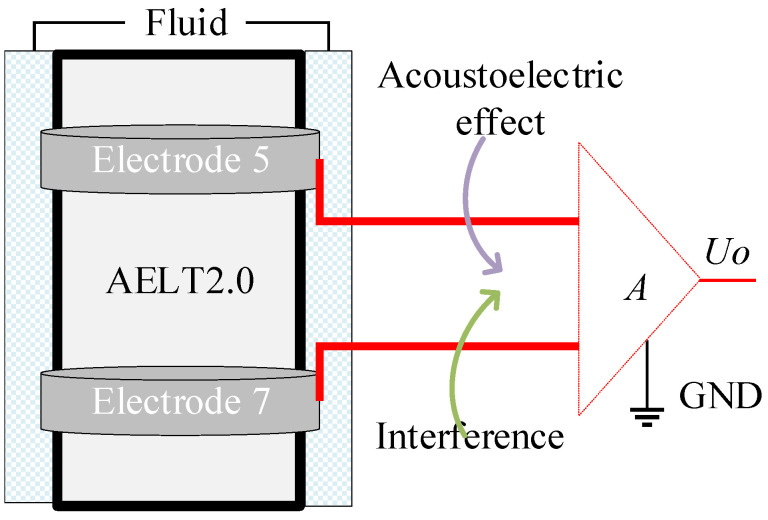
Schematic diagram of the electrodes and measurement circuits.

The acoustoelectric signals are weak. The detector is designed to comprise analogue signal processing and data acquisition circuits with a high signal-to-noise ratio and large dynamic range response characteristics. The gain dynamic range designed for each channel is 90 dB, and the passband range is (2~22) kHz. Figure 3 is a schematic diagram of the electrodes and their measurement circuit. The electrode input circuit uses a two-core shielded line, with the signal line connected to the measuring electrodes and the shielded casing connected to the signal ground.

### 2.4. Qualitative Electrical Interference Model

**Figure 4:**
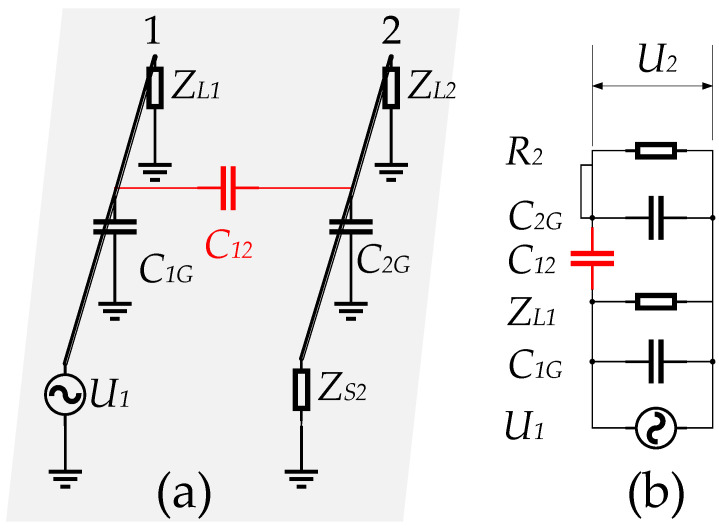
Model of capacitive coupling between two parallel conductors on the ground plane. (**a**) Coupled model; (**b**) Equivalent circuit.

High-voltage pulses interfere with the electrode measurement loop through capacitive coupling. The electric field interaction between two adjacent circuits is capacitive coupling, also known as electrical coupling [20]. Figure 4 shows the capacitive coupling model and its equivalent circuit between a pair of parallel wires on the ground. In Figure 4a, conductor 1 is the interference line. $U_{1}$ is the interference source. $Z_{L1}$ is the termination load. Wire 2 is the victim wire. $Z_{S2}$ and $Z_{L2}$ is the termination load. $C_{12}$ is the distributed capacitance between conductors 1 and 2. $C_{1G}$ and $C_{2G}$ are the distributed capacitances between the wires 1 and 2 and the ground, respectively.

According to the equivalent circuit in Figure 4b, the interference voltage on circuit 2 can be expressed as:

(1) $$U_{2}=\frac{j\omega C_{12}R_{2}}{1+j\omega R_{2}\left(C_{12}+C_{2G}\right)}U_{1}$$

where $R_{2}$ is defined as $R_{2}=Z_{S2}∥Z_{L2}$.

If $R_{2}$ is a low impedance and satisfies:

(2) $$R_{2}\leq \frac{1}{j\omega \left(C_{12}+C_{2G}\right)}$$

Then the expression for $U_{2}$ can be reduced to

(3) $$U_{2}\approx j\omega C_{12}R_{2}U_{1}$$

The small internal resistance of the signal between the fluid and the electrodes satisfies the assumption that $R_{2}$ is low impedance. The interference voltage $U_{2}$ is 90 degrees ahead of the phase of the interference source $U_{1}$. Once the detector system is established, the distributed capacitance $C_{12}$ is fixed and the interference voltage $U_{2}$ is mainly positively related to the resistivity $R_{2}$ and the frequency ω. The higher the energy of high frequency ω interference, the more serious the distortion of $U_{2}$.

### 2.5. Qualitative Electrical Interference Experiment

**Figure 5:**
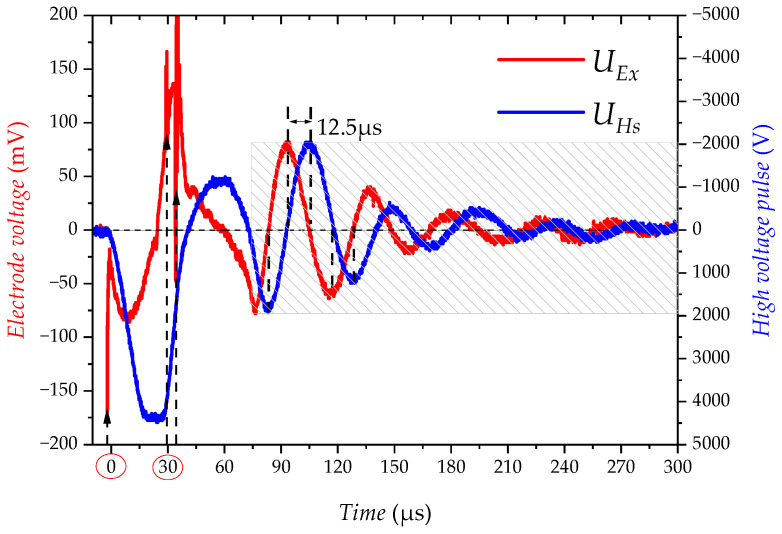
Time domain diagram of $U_{Ex}$ and $U_{Hs}$ (experiment).

The following shows the experimental results of the main interference cause analysis of the acoustoelectric detector, not the interference test results of AELT 2.0. As shown in Figure 5, a very strong high-frequency spike pulse is generated at the moment when the switching tube Q turns on and off (the position indicated by the black arrow). The energy of the high-frequency interference causes a brief distortion of the electrical interference. The electrical interference $U_{Ex}$ has the same pattern as the high-voltage pulse $U_{Hs}$, but the electrical interference $U_{Ex}$ is 90 degrees ahead of the phase of the high-voltage pulse $U_{Hs}$ (shaded area shown in the figure). The experimental results are in high agreement with the qualitative electrical interference model.

**Figure 6:**
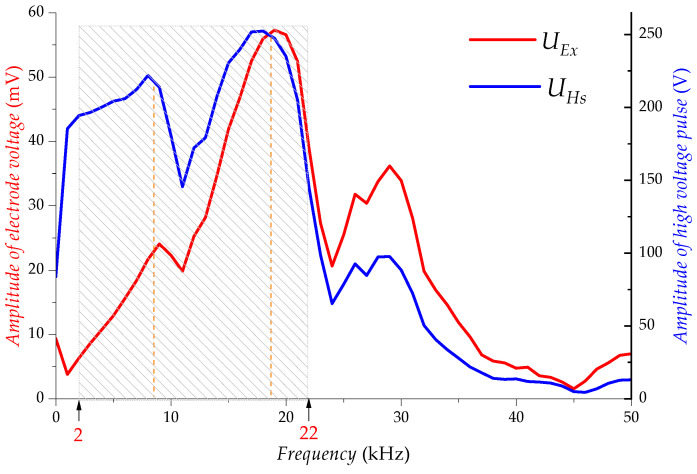
Frequency domain diagram of $U_{Ex}$ and $U_{Hs}$ (experiment).

As shown in Figure 6, the frequency-amplitude characteristics of the electrical interference $U_{Ex}$ and the high-voltage pulse $U_{Hs}$ match extremely well in the passband range of (2~22) kHz, which indicates that the interference voltage $U_{Ex}$ on the electrodes is due to the electrical coupling of the high-voltage pulse $U_{Hs}$.

## 3. Electrical Interference Simulation and Prediction Model

### 3.1. Equivalent Circuit for Electrode Measurement

**Figure 7:**
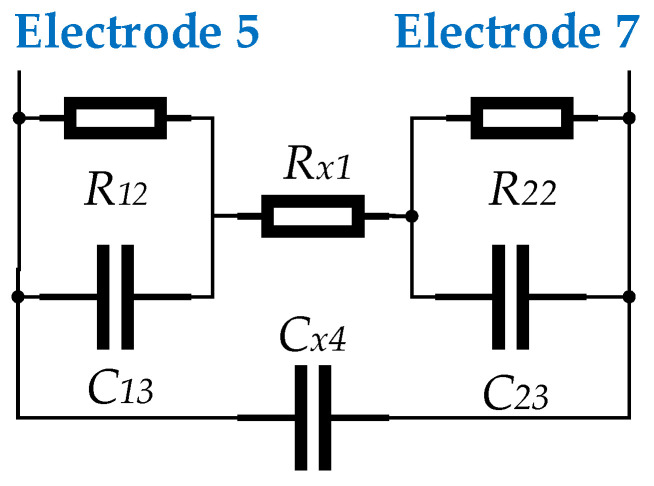
Equivalent circuit composed of electrode and fluid.

The electrode equivalent circuit [21] are shown in Figure 7. $R_{12}$ and $R_{22}$ are the contact resistance formed by the two measuring electrodes in contact with the fluid respectively. $C_{13}$ and $C_{23}$ are the double layer capacitance formed by the two measuring electrodes in contact with the fluid respectively. $C_{x4}$ is the capacitance between the two measuring electrodes. $R_{x1}$ is the resistance of the fluid. The signal internal resistance of the sensor is mainly the $R_{12}$, $R_{22}$ and $R_{x1}$. Higher signal internal resistance can introduce greater interference and at the same time can destroy the amplification characteristics of the preamplifier.

**Figure 8:**
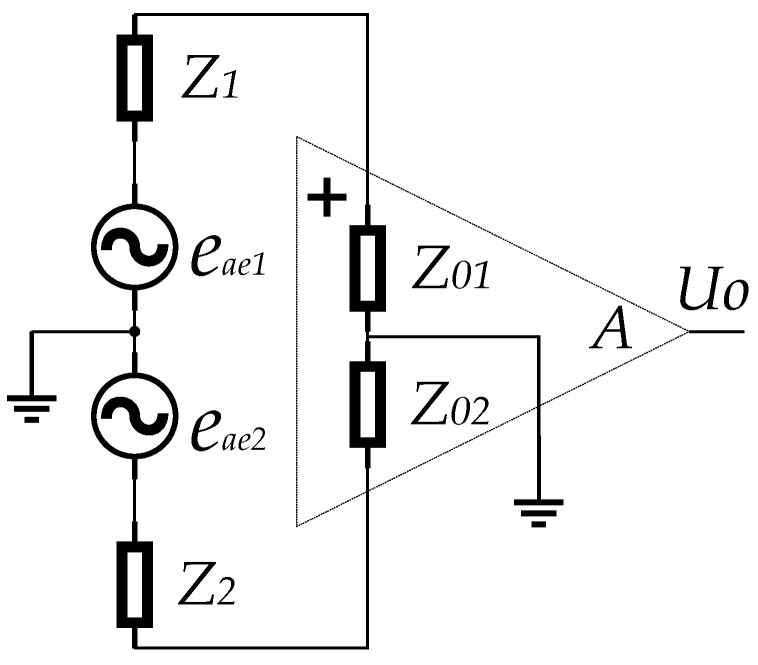
Equivalent circuit diagram of the detector.

The equivalent circuit diagram of the electrode measurement structure of the detector are shown in Figure 8. Both the circuit and the signal can be expressed in symmetric form and the parameters of symmetry are assumed to be equal. The corresponding measurement relationship is as follows:

(4) $$U_{o}=A(e_{ae1}\frac{Z_{01}}{Z_{1}+Z_{01}}+e_{ae2}\frac{Z_{02}}{Z_{2}+Z_{02}})$$

where $e_{ae1}$ and $e_{ae2}$ are the acoustoelectric signal voltage. A is the amplifier gain. $U_{o}$ is the amplifier output. $Z_{1}$ and $Z_{2}$ are the equivalent impedance of the fluid between the electrodes. $Z_{01}$ and $Z_{02}$ are the equivalent input resistances of the amplifier input.

The interference signal picked up by the electrodes is introduced passively but is analyzed here as a measurement signal. Assuming that the interference signal picked up by the electrode is $U_{Ex}$, the acoustoelectric signal picked up by the electrode is $U_{Ae}$, and the total signal picked up by the electrode is $U_{57}$, the measurement equation is therefore as follows:

(5) $$U_{57}=\left(U_{Ae}+U_{Ex}\right)$$

The equivalent circuit of the electrode measurement loop based on Equation (5) is built to analyze the quantitative relationship between the acoustoelectric signal $U_{Ae}$ and the interference signal $U_{Ex}$ in the electrode measurement loop. The electrical interference applied to the electrode by the interference source can be equated to an electrical excitation acting on the impedance formed at both ends of the electrode.

### 3.2. Equivalent Circuit for Electrode Measurement Based on Electrical Coupling

**Figure 9:**
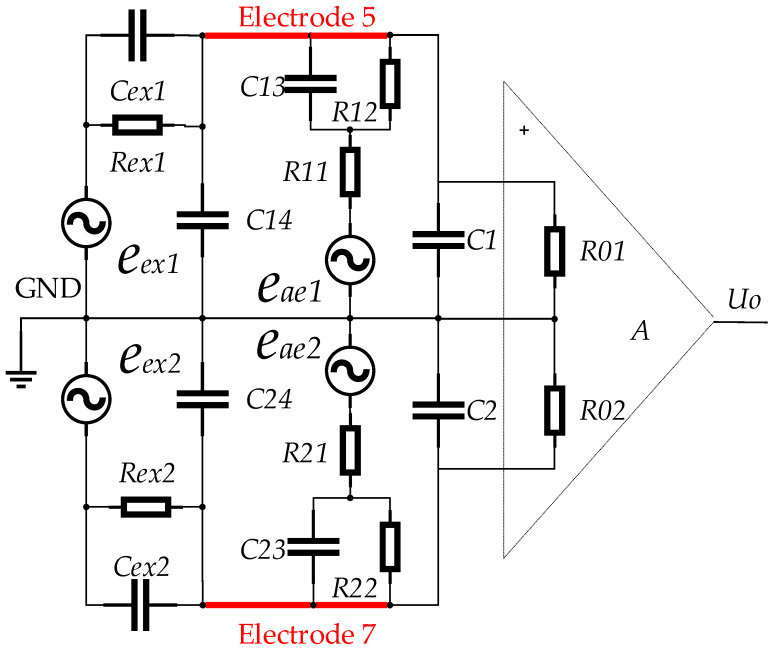
Equivalent circuit of electrode measurement circuit based on electric coupling.

The interference source acts on the electrode measurement loop through electrical coupling, so the equivalent circuit diagram of the electrode measurement loop based on electrical coupling is shown in Figure 9.

The equivalent circuit shown in Figure 9 is a symmetrical structure centered on the signal ground, and the symmetrical parameters are assumed to be equal. According to the characteristics and working principle of the measuring circuit, the description and relationship of each parameter in the figure are as follows:

A is the amplification factor of the pre-stage differential amplifier; $U_{o}$ is the output of the amplifier; $R_{01}$ and $R_{02}$ are the input resistance of the amplifier; $e_{ae1}$ and $e_{ae2}$ are the signal source of the acoustoelectric effect; $R_{11}$ and $R_{21}$ are the equivalent resistance of the fluid between the electrode and the signal ground; $R_{12}$ and $R_{22}$ are the polarization contact resistance between the electrode and the fluid; $C_{1}$ and $C_{2}$ are the capacitance between the measuring cable and the ground wire; $C_{13}$ and $C_{23}$ are the electric double layer capacitance formed by the contact between the electrode and the fluid; $C_{14}$ and $C_{24}$ are the capacitance between the two measuring electrodes; $e_{ex1}$ and $e_{ex2}$ are sources of interference; $R_{ex1}$ and $R_{ex2}$ are the coupling resistances of the interference source acting on the electrodes, which can be considered as the resistive part of the internal resistance of the signal source. $C_{ex1}$ and $C_{ex2}$ are the coupling capacitance of the interference source acting on the electrodes, which can be regarded as the capacitive reactance part of the internal resistance of the signal source.

The acoustoelectric signal picked up by the electrode satisfies the following relationship:

(6) $$U_{Ae}=\alpha (e_{ae1}+e_{ae2})$$

where α is the transmission coefficient of the acoustoelectric effect.

The coupling between the high voltage pulse and the electrode measurement loop is mainly through the distributed capacitance, since there is no specially designed coupling channel between them. For electrode measurement loops, high-voltage pulse sources are high-impedance signal sources because of the distributed capacitance between arbitrary conductors, whose magnitude is related to the distance and the surface area of the coupling between conductors, usually in the pF order [21]. From Figure 9, it can be seen that the model has negligible shunting effect on the acoustoelectric signal, although the equivalent impedance of the interference source constitutes a parallel relationship with the electrodes. The equivalent circuit for electrode measurements based on electrical coupling describes both the electrical coupling process from the interference source to the electrode and the process of picking up the acoustoelectric signal by the electrodes.

The interference signal picked up by the electrodes is expressed as follows.

(7) $$U_{Ex}=\beta \left(e_{ex1}+e_{ex2}\right)$$

where β is the transmission coefficient of electrical coupling.

The signal obtained at the output of the signal amplifier includes the acoustoelectric signal and the interference signal, so that the following measurement equation can be obtained.

(8) $$U_{o}=A\left(U_{Ae}+U_{Ex}\right)=A\left(\alpha \left(e_{ae1}+e_{ae2}\right)+\beta \left(e_{ex1}+e_{ex2}\right)\right)$$

### 3.3. Parameters of the Model

The transmission characteristics of the interference source in the electrode measurement loop are modeled and analyzed separately, since the electrode measurement loop is a linear signal transmission system.

(9) $$U_{Ex}=\beta \left(e_{ex1}+e_{ex2}\right)$$

**Figure 10:**
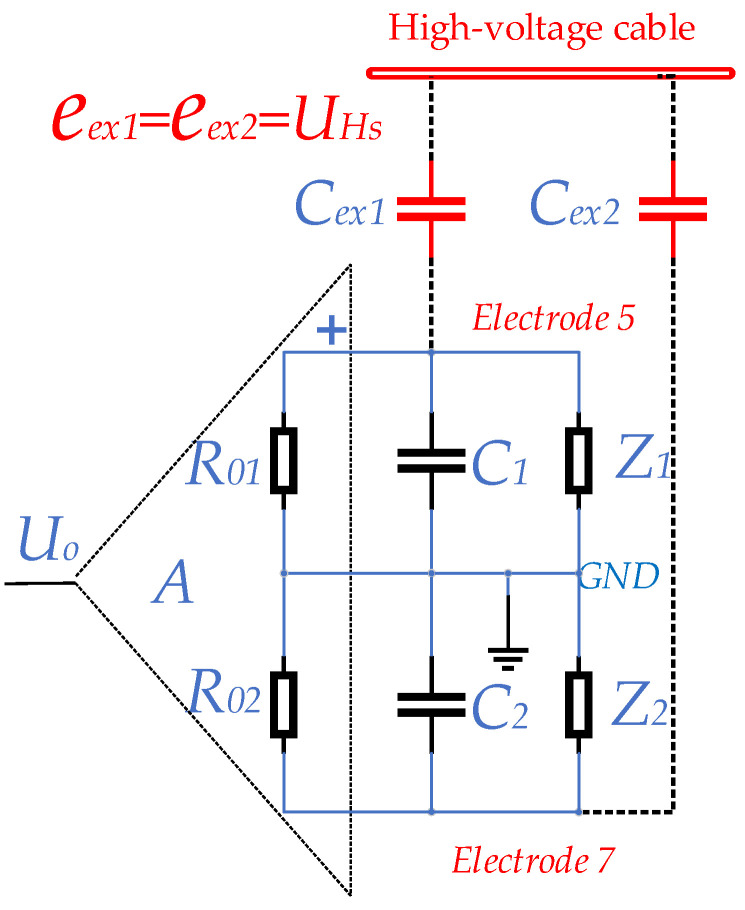
Equivalent Circuit of Coupling between High-voltage Cable and Electrode Loop through Distributed Capacitance.

The coupling path of the high-voltage cable to the electrode measurement loop is mainly the distributed capacitance $C_{ex1}$ and $C_{ex2}$, as its resistance path is mainly the leakage resistance, which can be seen as an open circuit, i.e., $R_{ex1}$ and $R_{ex2}$ are infinite. An equivalent circuit is formed as shown in Figure 10 with excitation sources $e_{ex1}$ and $e_{ex2}$.

In Figure 10, $C_{ex1}$ and $C_{ex2}$ are the distributed capacitances. $C_{1}$ and $C_{2}$ are the capacitance between the electrode signal line and the ground line. $Z_{1}$ and $Z_{2}$ are the equivalent impedances between the electrodes and the fluid, with polarization impedance. $R_{01}$ and $R_{02}$ are the equivalent input resistances of the two input ends of the amplifier, respectively.

Let the radius of the metal wire of both the measuring electrodes and the high-voltage cable be r. The distance between the two measuring electrodes and the high-voltage cable is $l_{1}$ and $l_{2}$, respectively, and the unshielded length is $l_{3}$. The distance between the two measuring electrodes and the high-voltage cable is expressed by the following relationship.

(10) $$l_{2}=l_{1}+0.2$$

Analyzing with the model of parallel wires [21], the distributed capacitance per unit length between two parallel wires can be found by the following equation.

(11) $$C=\frac{\pi \varepsilon }{\ln\frac{l}{r}}$$

where l is the distance between the wires, r is the radius of the wires, π is the circumference ratio, and the dielectric constant of the medium is ε.

**Figure 11:**
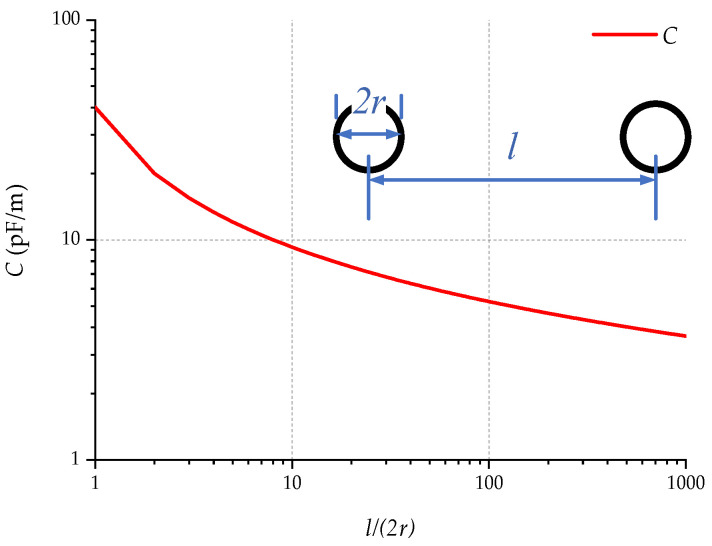
Relationship between the distributed capacitance of a parallel wire and its geometry (ε=1).

When the intermediate medium is air, the above equation is used to obtain the theoretical value of the distributed capacitance between the parallel lines. In practice, the distributed capacitance between the lines can be calculated by finding the coefficients according to the geometry of the lines in Figure 11.

Depending on the structure of the detector, the parameters are taken as follows: $l_{1}=5\;\mathrm{mm}$, $l_{2}=205\;\mathrm{mm}$, $l_{3}=20\;\mathrm{mm}$, and r=0.5 mm. Calculate the distributed capacitance $C_{ex1}$ and $C_{ex2}$.

(12) $$C_{ex1}\approx 40\;\mathrm{pF}/m\times 0.02\;m=0.8\;\mathrm{pF}$$

(13) $$C_{ex2}\approx 6\;\mathrm{pF}/m\times 0.02\;m=0.12\;\mathrm{pF}$$

The signal line is a twisted pair with the shield connected to the signal ground, and its length is taken as 1.5 m. Assuming that the ratio of the diameter of the signal line to the distance between the two wires is 2, the distributed capacitance between the signal line and the signal ground is obtained:

(14) $$C_{1}\approx C_{2}=11\;\mathrm{pF}/m\times 1.5\;m=16.5\;\mathrm{pF}$$

The actual capacitance will be greater than the above value because the intermediate medium is an insulating plastic, whose dielectric constant is usually greater than 1, for example, the commonly used polypropylene has a dielectric constant of about 3. Here we take $C_{1}\approx C_{2}=150\;\mathrm{pF}$.

### 3.4. Construction of the Model

A simulation and prediction model of electrical interference based on the logging environment was developed, since changes of $Z_{1}$ and $Z_{2}$ in the logging environment can have an impact on the electrode measurements.

The AC/DC module of Finite Element Software is used for the simulation study because the finite element method can simulate complex detector structures as well as logging environments and the current mode of the AC/DC module is suitable for quasi-static field time harmonic analysis with small current conduction and dielectric materials [22].

**Figure 12:**
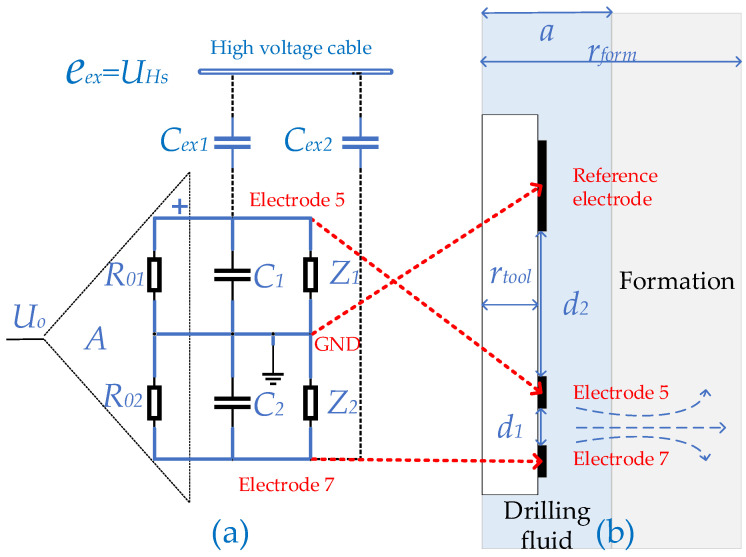
Electrically coupled model. (**a**) Signal model; (**b**) Formation model.

Based on the structure of AELT 2.0 and the logging environment, a two-dimensional axisymmetric model was developed, as shown in Figure 12. The ring-shaped differential electrode pairs (E5, E7) and the reference electrode, all arranged at the outer surface of the detector. The distance $d_{1}$ between E5 and E7 is 0.2 m. The distance $d_{2}$ between E5 and the reference electrode is 3.5 m. The radius of the detector $r_{tool}$ is 45 mm, the radius of the borehole a is 100 mm, and the radius of the formation $r_{form}$ is 2 m. Assume that the conductivity of the drilling fluid $\sigma_{m}$ is 1 S/m and the conductivity of the formation $\sigma_{t}$ is 0.1 S/m.

In Finite Element Software, the equivalent circuit for electrode measurements based on electrical coupling is coupled to the “current” field via the “circuit” interface. The parameter values of Figure 12 are as follows: $R_{01}=R_{11}=100\;k\Omega$, $C_{01}=C_{11}=20\;\mu F$, $C_{1}\approx 0.8\;\mathrm{pF}$, $C_{2}≃0.12\;\mathrm{pF}$, and $C_{1}\approx C_{2}=150\;\mathrm{pF}$. The expression for the high-voltage pulse $U_{Hs}$ is as follows:

(15) $$U_{Hs}\left(t\right)=6000e^{-20859.5t}\sin\left(1.1\times 10^{5}t\right)$$

### 3.5. Results of Simulation and Prediction

**Figure 13:**
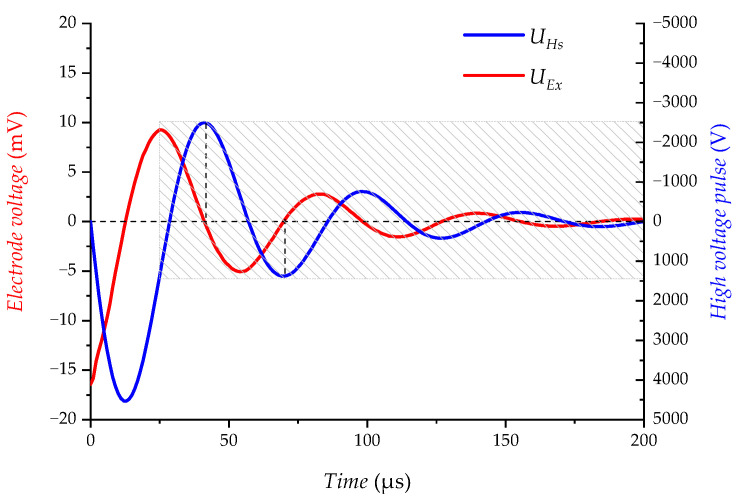
Time domain diagram of $U_{Ex}$ and $U_{Hs}$ (simulation).

Figure 13 shows the time domain simulation of the $U_{Ex}$ and the $U_{Hs}$. The simulation results are consistent with the qualitative analysis results in Section 2.4. The electrical interference has the same pattern as the high-voltage pulse (shaded area), but the electrical interference is 90 degrees ahead of the phase of the high-voltage pulse. At the moment 0, the electrode receives an interference signal of about a dozen millivolts, which is characterized by a rapid oscillatory decay. The interference signal magnitude and characteristics obtained from the simulation are similar to the interference test results of AELT 2.0, which shows that the selection of the detector system parameters is reasonable. According to the detection characteristics of AELT 2.0, the interface acoustoelectric conversion wave acquired by the electrode is in the mV to sub-mV range, and its arrival time is very early, about 10 μs. At this time, the amplitude of the electrical interference is greater than the interface acoustoelectric conversion wave, which is very unfavorable to the measurement of the interface conversion wave at zero source distance.

**Figure 14:**
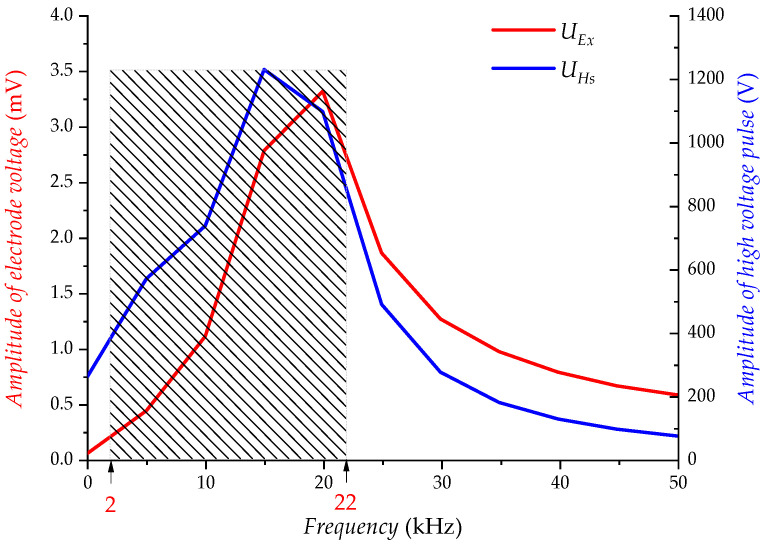
Frequency domain diagram of $U_{Ex}$ and $U_{Hs}$ (simulation).

Figure 14 shows the frequency domain simulation of the $U_{Ex}$ and the $U_{Hs}$. The frequency-amplitude characteristics of the electrical interference $U_{Ex}$ and the high-voltage pulses $U_{Hs}$ match very well, indicating that the frequency components of the electrical interference originate from the high-voltage pulses. The main component of the electrical interference falls in the frequency band range of (2~22) kHz, because High voltage pulses with high power excite the transmitting transducer to produce acoustic waves while also excite strong electrical interference.

Therefore, the interference signal not only affects the observation of the acoustoelectric signal, but it also leads to the inability to obtain clean results by data processing.

**Figure 15:**
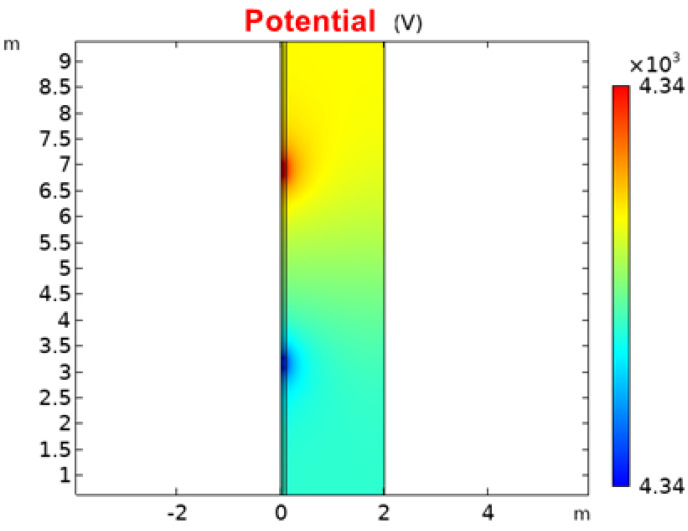
Potential distribution of the borehole and the formation at 10 μs.

Figure 15 shows the potential distribution of the borehole and the formation at the 10 μs moment. The detection range of the electrodes is large enough to reflect the contribution of the formation. The formation radius of 2 m is still used for the subsequent simulations, because the effect of the formation beyond 1 m on the measurement results is small according to the potential distribution.

**Figure 16:**
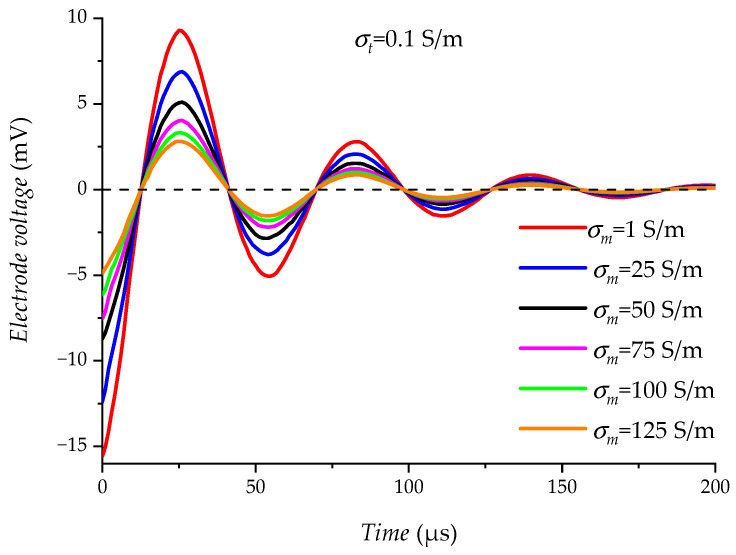
Relationship between $\sigma_{m}$ and $U_{Ex}$.

The effect of the change in drilling fluid conductivity $\sigma_{m}$ on the electrode measurement results is examined in the range of (1∼125) S/m, when the formation conductivity $\sigma_{t}$ is 0.1 S/m. As shown in Figure 16, the electrical interference $U_{Ex}$ decreases as the drilling fluid conductivity $\sigma_{m}$ increases. Large changes in drilling fluid conductivity $\sigma_{m}$ do not have a significant effect on electrical interference $U_{Ex}$ because of the deeper detection range of the electrodes.

**Figure 17:**
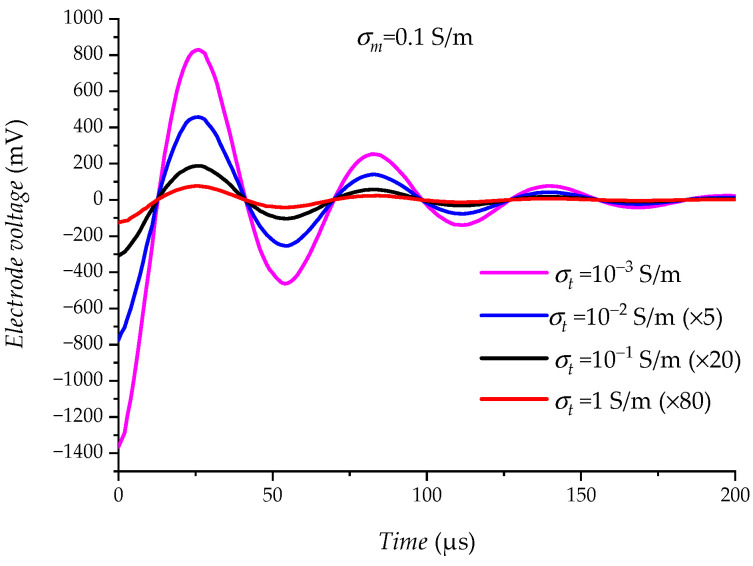
Relationship between $\sigma_{t}$ and $U_{Ex}$. (The curves are moderately enlarged for ease of display).

The effect of the change in formation conductivity $\sigma_{t}$ on the electrode measurement results is examined in the range of $(10^{-3}∼1)\;S/m$, when the drilling fluid conductivity $\sigma_{m}$ is 0.1 S/m. As shown in Figure 17, the electrical interference $U_{Ex}$ decreases as the formation conductivity $\sigma_{t}$ increases. Large changes in formation conductivity $\sigma_{t}$ have a greater impact on electrical interference $U_{Ex}$ because of the deeper detection range of the electrodes.

Electrical interference is positively correlated with the resistivity of the electrode measurement environment, which is consistent with the qualitative analysis in Section 2.4.

## 4. Conclusions

High-voltage pulses generate interference signals to the electrode measurement loop through capacitive coupling. In this paper, an equivalent model of electrode measurement based on electrical coupling is constructed on the basis of qualitative analysis of the causes of interference. The distributed capacitance of the model is calculated according to the system characteristics of AELT 2.0. The model portrays the logging environment in detail, as resistivity changes in the electrode measurement environment can have an impact on the model results. Ultimately, an electrical interference simulation and prediction model of the acoustoelectric logging detector is formed.

The quantitative simulation results for electrical interference and high voltage pulses are consistent with the qualitative analysis of the interference characteristics. 1. The electrical interference has the same shape as the high-voltage pulse, but the electrical interference is 90 degrees ahead of the phase of the high-voltage pulse. 2. The frequency-amplitude characteristics of electrical interference originate from high-voltage pulses.

The predicted results of electrical interference in the logging environment are as follows. 1. the magnitude of electrical interference is proportional to the formation resistivity. 2. large changes in formation resistivity have a large effect on electrical interference.

The direction of interference suppression is proposed: Electrical interference is the main factor because the transmitting transducer is close to the electrode. Isolation of the coupling path by means of an electrical shield is the most effective method. The shield plays the role of reducing the distributed capacitance between the high-voltage pulse and the electrode measurement loop.

## Data Availability

The study did not report any data.
